# Efficacy of Vitamin E in Methotrexate-Induced Hepatotoxicity in Rheumatoid Arthritis: An Open-Label Case-Control Study

**DOI:** 10.1155/2020/5723485

**Published:** 2020-05-01

**Authors:** Binit Vaidya, Manisha Bhochhibhoya, Shweta Nakarmi

**Affiliations:** National Center for Rheumatic Diseases (NCRD), Kathmandu, Nepal

## Abstract

**Objective:**

To examine the efficacy of vitamin E in methotrexate- (MTX-) induced transaminitis in patients with rheumatoid arthritis (RA).

**Methods:**

A case-control study was conducted at a tertiary rheumatology center for 12 months. Patients with RA on MTX and deranged aminotransferases were included. Patients with previous liver diseases, baseline transaminitis before methotrexate initiation, alcohol intake, muscle diseases, under hepatotoxic drugs, and *aminotransferases* > 3 times the upper normal limit were excluded. The patients were divided into treatment (vitamin E 400 mg bid for 3 months) and control groups (no vitamin E) using a random number table. The dose of MTX was unaltered. Follow-up was done after 3 and 6 months. Independent *t*-test was done to compare means of two groups. Paired *t*-test was done to compare differences in mean.

**Results:**

Among 230 patients, 86.5% were female with a mean BMI of 25.9 ± 4.5 kg/m^2^. In the treatment group, SGPT and SGOT at baseline were 73.1 ± 20.4 and 60.2 ± 24.5 IU/L, respectively; at 3-month follow-up 44.6 ± 34.2 and 38.3 ± 20.8 IU/L, respectively; and at 6-month follow-up 40.4 ± 35.7 and 34.2 ± 21.9 IU/L, respectively. In the control group, SGPT and SGOT at baseline were 63.4 ± 15.1 and 46.8 ± 13.7 IU/L, respectively, and at 3-month follow-up 55.8 ± 45.9 and 45.5 ± 30.9 IU/L, respectively. Significant decrease in the level of aminotransferases was seen in the treatment group (*p* value < 0.001) and not in the control group (*p* values 0.161 and 0.728, respectively). The change in levels of SGPT and SGOT from baseline to 3 months of follow-up was statistically significant in between two study groups (*p* values 0.007 and <0.001, respectively). From the control group, 29 patients were crossed over to vitamin E for the next 3 months. SGPT and SGOT decreased from 97.6 ± 44.1 to 46.1 ± 40.9 and 69.3 ± 34.9 to 29.1 ± 11.6 IU/L, respectively (*p* values 0.031 and 0.017, respectively).

**Conclusion:**

Vitamin E significantly attenuates MTX-induced transaminitis.

## 1. Introduction

Methotrexate (MTX) is a folate antagonist which inhibits dihydrofolate reductase (DHFR) [1]. It is considered a sheet anchor drug in the management of rheumatoid arthritis (RA) [2]. Low-dose MTX in weekly regime has been used as either monotherapy or in combination with other disease-modifying drugs for the past 3 decades [3]. It has transformed the management of RA by altering the course of disease, improving the patient's quality of life, and decreasing disease-related morbidity [4, 5]. However, it is associated with numerous side effects, common ones being gastrointestinal adverse effects, hepatotoxicity, stomatitis, alopecia, and hematological abnormalities [6, 7]. Liver abnormalities ranging from asymptomatic transaminase elevations to fibrosis and fatal hepatic necrosis can be associated with MTX [8]. Elevated transaminase is one of the most common adverse reactions seen with the use of low-dose MTX with a cumulative incidence of 48.9% and elevation above twice the upper limit of normal (UNL) in 16.8% leading to either discontinuation or dose reduction [9, 10]. With proper monitoring and treatment regimes, the incidence has decreased in current years to 22%, with less than 1% developing transaminitis more than twice the UNL [9].

Concurrent use of folic acid at the dose of 1 mg per day is recommended to reduce MTX toxicity, including hepatotoxicity. In case of transaminitis, monitoring of serum aminotransferases is done on a monthly basis with ongoing MTX treatment and withdrawal of MTX is recommended if the serum aminotransferase level is more than three times the UNL [11] or persistently above twice the UNL for more than 1 month [12].

The usage of natural compounds or drugs, nontoxic cytoprotective agents like ursodeoxycholic acid, *β*-carotene, etc. as adjuvants may play a significant role in decreasing the incidence of side effects of MTX [13, 14]. Among various adjuvant agents, vitamin E or alpha-tocopherol has reconstructing and antioxidant effects [15]. Pretreatment with vitamin E had shown protective effects in the intestinal tissue of MTX-treated rats [16]. Also, it was effective in the prevention of hepatotoxicity induced by MTX in the animal model [17]. But limited studies have been done showing the effectiveness of vitamin E supplementation in the treatment of hepatotoxicity induced by MTX in humans.

Thus, this study is aimed at observing the efficacy of vitamin E in MTX-induced hepatotoxicity in RA patients.

## 2. Materials and Methods

### 2.1. Patient Selection

This was a prospective, randomized, open-labeled, case-control study on the patients with RA conducted at the National Center for Rheumatic Diseases (NCRD), Kathmandu, Nepal, for 6 months. The ethical clearance for the study was obtained from the Nepal Health Research Center (NHRC), Nepal. Patients aged ≥18 years presenting to the rheumatology outpatient department and diagnosed as having RA according to 2010 American College of Rheumatology (ACR)/European League against Rheumatism (EULAR) classification criteria for RA [18] under MTX treatment and deranged aminotransferases between 1 and 3 times the UNL were selected. Patients with previous liver diseases including nonalcoholic fatty liver disease, deranged transaminases of unknown cause at baseline, and muscle diseases; patients under hepatotoxic drugs; alcoholics; and patients with *aminotransferases* > 3 times the UNL were excluded. Informed written consent was taken from the candidates who met the criteria.

### 2.2. Randomization

Convenient sampling technique was used where patients with rheumatoid arthritis visiting the rheumatology clinic at NCRD were recruited. Randomization was done with the help of a random number table generated online. Each patient was then assigned to either treatment or control group. Both physician and patient were aware of the treatment received.

### 2.3. Treatment Protocol

The treatment protocol for the treatment group is as follows: oral MTX (7.5-20 mg) weekly (continuation of patient's ongoing dose) and folic acid (1 mg daily) with vitamin E 400 mg twice a day along with advice on reduced fat and carbohydrate diet and aerobic exercises for at least 30 minutes a day for at least 5 days a week for 3 months. Vitamin E was given at a dose of 800 mg daily (in divided doses) as it was the most commonly used regime for nonalcoholic steatohepatitis [19, 20] with acceptable drug safety. If the level of transaminases remained high (more than 1-fold and less than 3-fold) at 3-month follow-up, the dose of methotrexate was reduced. If the level increased to more than 3-fold, then methotrexate was discontinued.

The treatment protocol for the control group is as follows: oral MTX (7.5-20 mg)/week (continuation of patient's ongoing dose) and folic acid (1 mg daily) along with advice on reduced fat and carbohydrate diet and aerobic exercises for at least 30 minutes a day for at least 5 days a week for 3 months. If the level of transaminases remained high (more than 1-fold and less than 3-fold) at 3-month follow-up, vitamin E was added in the abovementioned protocol (crossover group) and patients were followed up after 3 months.

### 2.4. Follow-Up Protocol

A dedicated research officer recorded the sociodemographic and clinical profiles at baseline and follow-ups (3 months and 6 months) in a predesigned excel sheet. C-reactive protein (CRP, mg/L; turbidimetry), erythrocyte sedimentation rate (ESR, mm/h; Westergren's method), complete blood count, kidney function test, and liver function tests (SGPT and SGOT) were done at baseline and subsequent follow-ups. SGPT and SGOT were measured using the International Federation of Clinical Chemistry (IFCC) recommendations without pyridoxal phosphate activation method [21] and expressed in IU/L. The normal reference ranges of SGPT and SGOT are 5-42 IU/L and 5-37 IU/L, respectively.

### 2.5. Quality Control Measures

Similar dietary and lifestyle advice was given to patients of both the groups. The patients in the treatment group were also asked to fill a drug diary on a daily basis and submit empty foils of vitamin E capsule on follow-up to ensure good compliance of the drug. All blood samples for transaminases were tested in the same laboratory with a fully automatic biochemistry analyzer (Erba Lachema XL 200, Czech Republic).

### 2.6. Statistical Analysis

Statistical analysis was done using SPSS 21 (IBM Corporation, USA). Simple descriptive statistics were used to describe baseline parameters. Mean, standard deviation, frequency, and percentages were used where applicable. Paired *t*-test was used to assess the difference in means of each group at baseline and follow-ups. Independent *t*-test was done to compare means between two groups. The *p* value of <0.05 was considered significant.

## 3. Results

A total of 230 patients were enrolled in the study. Among them, 86.5% were female, majority being housewives (69.5%). The mean age of the participants was 47.3 ± 10.7 years with a mean BMI of 25.9 ± 4.5 kg/m^2^. The most frequent (47.0%) dose of MTX where transaminitis occurred was 20 mg per week. Other sociodemographic and clinical profiles are shown in Table 1.

In the treatment group, vitamin E was administered in 106 patients. At baseline, the mean SGPT and SGOT levels were 73.1 ± 20.4 and 60.2 ± 24.5 IU/L, respectively. There was a statistically significant decrease in the levels of SGPT and SGOT to 44.6 ± 34.2 and 38.3 ± 20.8 IU/L, respectively (*p* value < 0.001) at 3 months. (Table 2). The change in mean SGPT from baseline to 3-month follow-up in treatment and control groups was 28.5 ± 41.8 and 7.5 ± 48.5 IU/L, respectively, and the change in mean SGOT in treatment and control groups was 21.8 ± 34.4 and.

1.2 ± 33.7, respectively. The change in levels of SGPT and SGOT from baseline to 3-month follow-up was statistically significant in between two study groups (*p* value 0.007 and <0.001, respectively) (Table 3). At 6-month follow-up, the SGPT and SGOT levels were 40.4 ± 35.7 and 34.2 ± 21.9, respectively.

In the control group, one hundred and twenty-four (124) patients were maintained on the same dose of MTX and were followed up at 3 months after dietary and exercise advice. The mean SGPT and SGOT levels at baseline were 63.4 ± 15.1 and 46.8 ± 13.7 IU/L, respectively, and at 3-month follow-up were 55.8 ± 45.9 and 45.5 ± 30.9, respectively. The decline was not statistically significant for SGPT and SGOT at 3-month follow-up (*p* value 0.161 and 0.728, respectively) (Table 2).

### 3.1. Crossover Group

In a subsequent follow-up, 29 patients from the control group were switched to the treatment group as the serum transaminase level increased from baseline. The SGPT and SGOT levels increased from 63.9 to 97.6 and 43.7 to 69.3 IU/L, respectively. After vitamin E supplementation at a dose mentioned for the treatment group, the level of serum SGPT and SGOT decreased significantly at follow-up 3 months later (Table 4).

### 3.2. Side Effect Profile

Mild nausea was observed in 2 patients which subsided on its own without any active intervention. Only 1 patient complained of abdominal discomfort, for which pantoprazole 40 mg once daily was added for few days with positive response.

## 4. Discussion

The discovery of methotrexate has enabled the control of many immune-mediated inflammatory diseases like RA, which were once considered to have no treatment options [22, 23]. However, it comes with a significant toxicity profile, the most important being hepatotoxicity when used at a high-dose or low-dose continuous therapy [6, 7, 10]. With more understanding of the mechanism of methotrexate action, weekly therapy and the use of folate supplementation have significantly reduced the rates of such reported toxicities [11, 24].

The exact mechanism for methotrexate-induced liver injury is not known. Various experimental studies have postulated local folate depletion causing intracellular accumulation of adenosine and oxidative injury with increased susceptibility to reactive oxygen species as possible mechanisms. Histological studies have shown various stages of methotrexate hepatotoxicity [25, 26] progressing from steatosis to cell hypertrophy, fibrosis, and cirrhosis [8]. A study showed an increased oxidative injury as measured by markers of lipid peroxidation in rabbits treated with methotrexate. The same study also demonstrated a beneficial effect of coadministration of vitamin E in the prevention of methotrexate-induced oxidative injury [27]. This increase in oxidative stress induced by methotrexate, as measured by malondialdehyde (MDA) levels, nitrite and nitrate levels, and the activities of antioxidants like superoxide dismutase (SOD), catalase (CAT), and total antioxidant status (TAS), was also shown to be beneficial in inducing apoptosis in psoriatic patients [28]. We believe that the use of vitamin E, which has shown a beneficial effect in nonalcoholic steatohepatitis, at an early stage of transaminase elevation (which probably would reflect the stage of steatosis), can ameliorate methotrexate-induced liver injury.

Based on these theories, a few animal studies have tested various compounds with antioxidant properties in the prevention of methotrexate-induced liver injury. Animal studies have shown beneficial effects of *β*-carotene, melatonin, and metformin in methotrexate-induced hepatotoxicity [14]. In this study, we tried to examine the effect of vitamin E (*α*-tocopherol) in patients with elevated transaminase levels after methotrexate administration at a low dose for varying intervals because of its proven potent antioxidant effects. *α*-Tocopherol functions as a peroxyl radical scavenger thus having an important function of maintaining the integrity of long-chain polyunsaturated fatty acids in the membranes of cells and maintaining their bioactivity [29].

Our results showed that the improvement in transaminase levels was statistically significant in those treated with vitamin E without modifying the dose of methotrexate as compared to those who were just advised diet and weight control. Even though the baseline liver enzymes were higher in the treatment group, use of vitamin E reduced them to significantly lower levels than the control group. We went a little further to explore the effectiveness in a subgroup of patients in a control group whose transaminase level deteriorated on follow-up. An improved transaminase level in this subgroup of patients further strengthened our hypothesis. Patients with elevation more than three times the upper limit were excluded due to ethical reasons, but we believe that coadministration of vitamin E might help them to maintain their dose so that the clinical benefit is not lost.

## 5. Limitations of the Study

The study was not placebo-controlled, and both the patients and investigators were not blinded to the treatment groups. Also, the crossover was not preplanned for all patients in the control group. The histological findings cannot be interpreted with the current methodology to prove our hypothesis.

## 6. Conclusion

This study showed that vitamin E coadministration is promising in attenuating the hepatotoxic effects of methotrexate and thus helps to maintain the effective dose in patients. However, more extensive studies in humans with a large sample size and longer intervention period need to be conducted for a better understanding of vitamin E in RA patients under long-term MTX therapy.

## Figures and Tables

**Table 1:** Sociodemographic and clinical profiles of the study participants at baseline (*n* = 230).

Parameters	EVA group (*n* = 106) *n* (%) or mean ± SD	Non-EVA group (*n* = 124) *n* (%) or mean ± SD	*p* value^∗^
Age (years)	47.27 ± 11.2	47.31 ± 10.30	0.332
Gender			
Male	11 (10.4)	20 (16.1)	
Female	95 (89.6)	104 (83.9)	
BMI (kg/m^2^)	26.3 ± 4.9	25.5 ± 4.2	0.291
Occupation			
Housewife	79 (74.5)	85 (68.5)	
Service holder	8 (7.5)	14 (11.3)	
Others	19 (18.0)	25 (20.2)	
Education			
Illiterate	23 (21.7)	17 (13.7)	
Can sign only	21 (19.8)	27 (21.8)	
Primary	6 (5.7)	10 (8.1)	
Secondary	28 (26.4)	39 (31.4)	
Higher secondary and above	28 (26.4)	31 (25.0)	
Disease duration in months	37.15 ± 31.32	43.80 ± 62.32	0.197
MTX dose (mg per week)	16.7 ± 3.8 (median, 15; mode, 20)	17.1 ± 3.2 (median, 15; mode, 20)	0.215
Enthesitis^#^	43 (40.6)	55 (44.4)	
Red eye	21 (19.8)	21 (16.9)	
Rheumatoid nodules	1 (0.9)	1 (0.8)	
Rheumatoid factor (IU/mL)	78 (76.5)	96 (77.4)	
ACPA (U/mL)	72 (67.9)	90 (72.6)	
CRP (mg/L) (median)	8.36 ± 13.99	6.69 ± 8.49	0.206
DAS 28	2.3 ± 0.9	2.2 ± 0.7	0.155
CDAI	6.1 ± 6.1	5.8 ± 5.1	0.329

BMI: body mass index; kg/m^2^: kilogram per square meter; ACPA: anticitrullinated protein antibodies; CRP: C-reactive protein; DAS 28: disease activity score for RA; CDAI: clinical disease activity index. ^∗^Independent *t*-test; ^#^enthesitis included history of enthesitis in the past or present and subclinical enthesitis or tenosynovitis detected on musculoskeletal ultrasonography.

**Table 2:** Comparison of transaminases levels at baseline and 3-month follow-up in treatment and control groups.

Groups (*n* = 230)	Baseline (IU/L)		3 month follow-up	*p* value^∗^
Treatment group (*n* = 106)	SGPT (IU/L)	73.1 ± 20.4	44.6 ± 34.2	<0.001
	SGOT (IU/L)	60.2 ± 24.5	38.3 ± 20.8	<0.001

Control group (*n* = 124)	SGPT (IU/L)	63.4 ± 15.1	55.8 ± 45.9	0.161
	SGOT (IU/L)	46.8 ± 13.7	45.5 ± 30.9	0.728

^∗^Paired *t*-test. SGPT: serum glutamic pyruvic transaminase; SGOT: serum glutamic-oxaloacetic transaminase.

**Table 3:** Comparison of change in transaminase levels from baseline to 3 months of follow-up in between two groups.

	Treatment group	Control group	*p* value^∗^
SGPT (baseline)	71.56 ± 21.78	64.22 ± 16.09	<0.001
*Δ* SGPT	28.5 ± 41.8 (median: 29.0)	7.5 ± 48.5 (median: 23.0)	0.007
SGOT (baseline)	61.01 ± 26.05	48.00 ± 15.08	<0.001
*Δ* SGOT	21.8 ± 34.4 (median: 18.5)	1.2 ± 33.7 (median: 11.0)	<0.001

^∗^ Independent *t*-test. *Δ* = baseline to 3 months of follow-up. SGPT: serum glutamic pyruvic transaminase; SGOT: serum glutamic-oxaloacetic transaminase.

**Table 4:** Comparison of transaminase levels at baseline and 3 months of follow-up in the crossover group (*n* = 29).

	Baseline	3-month follow-up	*p* value	6-month follow-up	*p* value^∗^
SGPT (IU/L)	63.9 ± 16.5	97.6 ± 44.1	0.010	46.13 ± 40.99	0.031
SGOT (IU/L)	43.7 ± 10.2	69.3 ± 34.95	0.007	29.13 ± 11.64	0.017

^∗^Paired *t*-test. SGPT: serum glutamic pyruvic transaminase; SGOT: serum glutamic-oxaloacetic transaminase.

## Data Availability

The data used to support the findings of this study are available from the corresponding author upon request.
